# An Iranian Consensus Document for Nutrition in Critically Ill Patients, Recommendations and Initial Steps toward Regional Guidelines

**Published:** 2017

**Authors:** Seyed Mohammadreza Hashemian, Robert G. Martindale, Hamidreza Jamaati, Ali Amirsavadkouhi, Salahaddin Mahmudi Azer, Mahdi Shadnoush, Seyed Hossein Ardehali, Atabak Najafi, Arezoo Ahmadi, Seyyed Reza Seyyedi, Ata Mahmoodpoor, Omid Moradi, Saeed Abbasi, Saeed Hosseini, Reza Shahrami, Saeed Abdi, Zahra Sepehri, Babak Omranirad, Seyed Amir Mohajerani, Pejman Rohani, Aliakbar Sayyari, Hossein Imani, Ali Akbar Velayati

**Affiliations:** 1 Clinical Tuberculosis and Epidemiology Research Center, National Research Institute of Tuberculosis and Lung Diseases (NRITLD), Shahid Beheshti University of Medical Sciences, Tehran, Iran,; 2 Oregon Health and Science University, Portland, OR,; 3 Chronic Respiratory Diseases Research Center, NRITLD, Shahid Beheshti University of Medical Sciences, Tehran, Iran,; 4 Urmia University of Medical Sciences, Urmia, Iran,; 5 Department of Clinical Nutrition, Faculty of Nutrition and Technology, Shahid Beheshti University of Medical Sciences, Tehran, Iran,; 6 Department of Critical Care, Shohadaye Tajrish Hospital, Shahid Beheshti University of Medical Sciences, Tehran, Iran,; 7 Department of Anesthesiology and Critical Care Medicine, Faculty of Medicine, Sina Hospital, Tehran University of Medical Sciences, Tehran, Iran,; 8 Lung Transplantation Research Center, Department of Cardiology, National Research Institute of Tuberculosis and Lung Diseases (NRITLD), Shahid Beheshti University of Medical Sciences, Tehran, Iran,; 9 Department of Anesthesiology, Faculty of Medicine, Tabriz University of Medical Sciences, Tabriz, Iran,; 10 Department of Anesthesiology and Critical Care, Iran University of Medical Sciences, Rassol-e-Akram Complex Hospital, Trauma and Injury Research Center, Iran University of Medical Sciences, Tehran, Iran,; 11 Anesthesiology and Critical Care Research Center, Isfahan University of Medical Sciences, Isfahan, Iran,; 12 School of Nutritional Sciences and Dietetics, Tehran University of Medical Sciences, Tehran, Iran,; 13 Anesthesiologist, Intensivist,; 14 Department of Gastroenterology, Shahid Beheshti University of Medical Sciences, Tehran, Iran,; 15 Department of Internal Medicine, Zabol University of Medical Sciences, Zabol, Iran,; 16 Department of Pediatric Gastroenterology, Hepathology and Nutrition, Mofid Children Hospital, Shahid Beheshti University of Medical Sciences, Tehran, Iran.

## INTRODUCTION

Short-term and prolonged metabolic stress seems to have major impact on critically ill patients. Indeed; the metabolic stress is known to increase both morbidity as well as mortality in Intensive Care Unit (ICU) patients.

Nutritional support can deliver energy and metabolic demands of critically ill patients in ICU(1). Nutrition support refers to enteral or parenteral provision of calories, protein, electrolytes, antioxidants, vitamins, minerals, trace elements, and fluids which may optimize recovery from critical illness(2). This support is aimed to meet energy demands and to avoid protein-energy deficit and consequent negative nitrogen balance(3). Indeed, malnutrition in critically ill patients is known to underline a number of potential complications including; hypercatabolism, impaired ventilatory drive, prolonged length of mechanical ventilation, increased septic complications, increased length of stay and even mortality rate(4–6).

The changes in metabolic pathways due to metabolic stress in critically ill patient result in a highly complex and dynamic metabolic state. This in turn leads to difficult, if not impossible, safe and effective management of nutrient in critically ill patient. In addition, the degree of existing malnutrition, the severity of illness, time of the initiation of nutritional support and adequacy of nutritional support, are known to affect outcomes in critical illness (7,8). However; a systematic review has shown that there are no specific and identical statistics on the effects of nutrition support on patients’ outcomes in intensive care unit, to this end nutritional support for critically ill patient in ICU remains a challenge for intensive care physicians (9).

Previous data shows that patient’s poor nutritional status is associated with increased length of hospital stay, as well as increased morbidity, mortality and medical expenses. It is also assumed that malnutrition is a prognostic factor for patients’ survival in critical illness. Malnutrition rate in general population has been estimated at 20–50% in the world with validation of similar statistics in Iranian studies (10,11). It is likely that the rate of malnutrition is higher in hospitalized patients. Indeed, malnutrition is estimated at 40–45% among patients with gastro-enteric diseases (12,13). Interestingly, a different Iranian Study has estimated the malnutrition at about only 11% among hospitalized patients (11). Perhaps, dissimilar patient populations, patient socioeconomic status, and variable definitions of malnutrition in may underline this wide range of difference in above mentioned studies. Thus; further studies and more regionally agreed upon, definition for malnutrition is needed to overcome the above mentioned discrepancy (14). It is likely that insufficient information among health care providers, regarding sufficient nutritional support, contributes to high rate of malnutrition among patients in Iranian health care facilities. Thus, providing regional guidelines may help to improve patients’ nutritional status and hence their general conditions and overall outcomes in Iranian Hospitals (11).

Current guideline for the Provision and Assessment of Nutrition Support Therapy in the Adult Critically Ill Patient have been determined by ASPEN which has discussed various aspects of nutrition in ICU, including; indications, time to initiate, rout of administration, dose of nutritional support, and monitoring of nutritional status (15). Given the variability of clinical practice and patient population in different parts of the world, it may not be feasible to use similar guidelines without regional adaptation and adjustments. The aim of the current document is to provide a simple and adaptable guideline for Iranian ICU environment.

## GUIDELINE RECOMMENDATIONS

### A. Nutrition Assessment and Screening

1. In ICU, the nutritional status assessment is essential to be part of daily care of critically ill patients. This indeed has been shown to reduce overall morbidity and mortality in this patient population (16).
2. Each patient admitted with critical illnesses should undergo initial nutritional status screening to identify patients with existing malnutrition or those at risk of malnutrition.
3. Accurate estimation of nutritional needs of critically ill patients in terms of calories and macronutrients, and ongoing assessment of above mentioned needs are recommended (17).
4. The amount of energy expenditure is directly reflected by the amount of energy released as heat (direct calorimetry) and indirectly reflected by the amount of oxygen consumption, CO2 production, and indirect calorimetry through urine urea nitrogen excreted.
5. The components of the daily Total Energy Expenditure (TEE) and Resting Energy Expenditure (REE) should be calculated based on following outlines.
6. Nutrition assessment should be carried out in the first 24 hours of admission and should be repeated a week later or periodically.
7. Anthropometric measurements including body weight, body mass index (BMI), mid-arm muscle circumference (MAMC), triceps skin-fold thickness (TSF), and calf circumference are better predictors of the nutritional outcome in critically ill patients than biochemical tests(18).
8. Routine monitoring of weight if possible is a valuable index of nutrition status in critically ill patients.
9. If ICU patients are exposed to lower energy intake compared to their requirements, their mid arm circumference is reduced significantly after 1 week(19).
10. Subjective global assessment (SGA) is a clinical questionnaire for bedside assessment of nutritional status based on the patient’s history and physical examination. This screening tool does not need any laboratory data. SGA has the most diagnostic value for critically ill patients among the different nutrition screening tools (20,21).
11. NUTRIC Score can be used to quantify the risk of critically ill patients developing adverse events that may be modified by aggressive nutrition therapy.

### Metabolic assessments

1. Nutrition assessment can be achieved by measuring the nitrogen balance and resting energy expenditure (REE).
2. Albumin, which has a large pool and much longer half-life (14–20 days), is not indicative of the immediate nutrition status.
3. High percentage of patients in ICU have low serum albumin levels, and the frequency of patients with low levels of albumin increase after seven days stay in the hospital(22).
4. Pre-albumin (also known as transthyretin or thyroxine-binding pre-albumin) is a stable circulating glycoprotein synthesized in the liver and has a half-life of 24–48 hours. It binds with retinol binding-protein and is involved in the transport of thyroxine as well as retinol. Pre-albumin serum concentration is diminished in liver disease and may be falsely elevated in renal failure.
5. C-reactive protein (CRP) as an index of the acute-phase response, when measured serially (once a day during the acute response period), is inversely related to serum pre-albumin. Decreases in serum CRP values to levels < 2 mg/dL have been associated with the return of anabolic metabolism.
6. Critically ill patients, anthropometric assessment is difficult to interpret because the measurement is influenced by factors caused by disease or trauma.

### Energy Requirement in the Critically Ill patients

To estimate energy requirement, Energy Expenditure (EE) should be calculated according to the following guideline:

1. *Basal energy expenditure* (BEE) also called *basal metabolic rate* (BMR) is the energy expended on awakening from a 12-hour fast measured in a thermoneutral environment (25°C). *Thermogenic effect of food i*s the energy expended after the ingestion of food. Energy expenditure may increase 5% to 10% after a meal.
2. *Resting energy expenditure* (REE) is the energy expended while resting in the supine position with open eyes. It includes the thermogenic effect of food if performed within a few hours of a meal or during continuous infusions of nutrients such as during continuous total parenteral nutrition (TPN) administration. REE is approximately 10% greater than BEE.
3. *Sleeping energy expenditure* (SEE) is the energy expended during sleep and is usually 10% to 15% lower than REE.
4. *Activity energy expenditure* (AEE) is the energy expended during physical activity. During maximum exercise, AEE can be 6- to 10-fold greater than the BEE.
5. *Total energy expenditure* (TEE) is the total energy expended over 24 hours; explicitly, TEE is the sum of the energy expended during periods of sleep, resting, and activity.
6. Energy expenditure increases to warm patients who are in a cold environment. Warming occurs through shivering and increased metabolism of brown fat (neonates).
7. Fever increases the metabolic rate about 10% per degree Celsius (C).
8. A simplistic weight-based equation (25–30 kcal/kg/day) could be used to determine energy requirements.

### Estimating Resting Energy Expenditure (REE)

1. To calculate REE using Harris-Benedict equation (Table 1) two types of factors should be accounted into the formula including stress factors and activity factors (Table 2). This formula is recommended for calculating required energy in ICU patients. In patients with significant illnesses, the altered metabolic milieu increases REE.
2. The Harris-Benedict equation estimated that patients with trauma, sepsis, chronic obstructive pulmonary disease (COPD), burns, and those who are spontaneously breathing after surgery, have REEs that are above the estimated REE (eREE).
3. REEs increase from 10–20% after elective surgery to 20–40% during sepsis.

**Table 1.** Harris-Benedict equation for Energy Expenditure and Caloric Requirements

**Harris-Benedict equation**
Males: eBEE (kcal/d) = 66 + (13.7 × W) + (5 × H) − (6.8 × A)
Females: eBEE (kcal/d) = 655 + (9.6 × W) + (1.7 × H) − (4.7 × A)
eREE = eBEE × stress factor
eTEE = eREE × activity factor

eBEE, Estimated basal energy expenditure; eREE, estimated resting energy expenditure; eTEE, estimated total energy expenditure

W, weight (kg); H, height (cm); A, age (years)

**Table 2.** Stress and activity factors for Harris-Benedict equation

**Stress factors**	
spontaneously breathing patients	Major surgery: 15%–25%
	Infection: 20%
	Long-bone fracture: 20%–35%
	Malnutrition: Subtract 10%–15%
	Burns: Up to 120%, depending on the extent
	Sepsis: 30%–55%
	Major trauma: 20%–35%
	COPD: 10%–15%
Mechanically ventilated patients	Subtract 10%–15%
**Activity factors**	
Sedated patients who are mechanically ventilated: 0%–5%	
Patients who are mechanically ventilated (assisted ventilation): 5%–10%	
Bedridden, unsedated patients who are spontaneously breathing: 10%–15%	
Patients who are sitting in a chair: 15%–20%	
Ambulating patients: 20%–25%	

COPD: Chronic obstructive pulmonary disease

### Measuring Energy Expenditure

Indirect calorimetry involves measuring oxygen consumption (VO_2_) and carbon dioxide production (VCO_2_) (Table 3). The gas exchange method is used to measure VO_2_ and VCO_2_, whereas the Fick method can be used to measure only VO_2_.

**Table 3.** Weir equation

**Energy expenditure (EE) (kcal/d)**	**1.44 (3.9 × VO2) + (1.1 × Vco2)**
REE	Measurements made at rest
TEE	REE × activity factor
TEE	Continuous measurement over 24 hours

### Measuring Nitrogen Balance

1. N balance = (N intake) − (N output); Negative balance = catabolism; Positive balance = anabolism
2. Where (N intake) = all protein/amino acid intake over 24 hours, whether enteral or parenteral
3. (N output) = (UUN + 4 + EL); UUN, Urine urea nitrogen over 24 hours; 4 g average fecal and cutaneous losses; EL, excessive losses, such as protein-rich drainages (e.g., pus).
4. 6.25 g protein/amino acids = 1 g nitrogen

Daily caloric requirements for ICU admitted patients are provided in Table 4.

**Table 4.** Daily Caloric Requirements

**Patients**	**Using Measured or eREE**	**Using Body Weight**
Sedated and mechanically ventilated	1.0 − 1.2 × REE	20–24 kcal/kg
Unsedated and mechanically ventilated	1.2 × REE	22–24 kcal/kg
Spontaneously breathing	1.2 − 1.3 × REE	24–26 kcal/kg
Spontaneously breathing (maintenance)	1.3 × REE	24–26 kcal/kg
Spontaneously breathing (repletion)	1.5 − 1.7 × REE	25–30 kcal/kg

### Management of nutritional support in ICU

1. Indications include prevention or treatment of malnutrition in ICU and supporting hypercatabolic patients such as those with sepsis or other critical illnesses.
2. Given malnutrition in preoperative period accompanies poor outcome, SIRS or postoperative infections, appropriate management of malnutrition in this patient population is indicated.
3. Nutritional support should be slowly initiated and increased only if the patient tolerates feeding.
4. TPN in perioperative period decreases postoperative infectious or non-infectious adverse effects.
5. If patient have bowel anastomosis or is not able to ingest for 7 days then TPN may be started from day 2.
6. In GI surgeries, if TPN is started during the first 24 hours of postoperative period, it would decrease risk of infection and dehiscence of anastomosis site but would increase risk of nausea and vomiting. While TPN is not routinely advised, the EN is.
7. Given patients in the ICU, who are under mechanically ventilated (MV) have a malnutrition rate of 38% to 100%(23), EN should be started in this patient population within first 48 hours. This has been shown to reduce infectious complications and shorten the duration of hospitalization,

### Parentral Vs Enteral Nutrition

1. The enteral route is preferred over the parenteral method because it is the natural port of entry for exogenous nutrients(24).
2. The first line recommended nutrition support is the early enteral nutrition (begin with 24–48 hours after admission), given it has been shown to reduce infectious risks and mortality in comparison with late EN and early parenteral nutrition (PN)(25,26). Thus early initiation of enteral feeding within 48 hours of ICU admission should be considered.
3. The gastro-intestinal adverse effects of not starting early EN include; vilous atrophy, decrease in epithelial cells and mucosal layer thickness, and increase in intestine permeability.
4. Parenteral nutrition should be used in patients at high nutritional risk when EN is not feasible or after the first week of hospitalization if EN is not sufficient(27).
5. Most patients are preferred to be initially fed via nasoenteric tube. In critically ill patients, NG tube should be placed distal to the ligament of Treitz due to high incidence of gasteroparesis.
6. Compared with gastric feedings, jejunal feedings facilitate higher caloric intakes. Passing the tube through the pylorus is often problematic because of gastroparesis as well as a supine and immobile state. Jejunostomy tube should be placed during surgery in patients not expected to eat soon after surgery (i.e., those with severe trauma, major head injury, or esophageal surgery).
7. Patients who will require prolonged enteral feeding (>4 weeks) should have either surgical gastrostomies/jejunostomies or percutaneously placement with endoscopic assistance.
8. Percutaneous endoscopic gastrostomies (PEGs) are commonly performed, and in a modified procedure a jejunal tube is placed through the PEG.
9. In critically ill patients, delivery of increased calories via the enteral route, with or without supplemental PN, is not associated with a survival benefit(28).

## FORMULATIONS

### Glucose and Carbohydrates

1. Unlike re-feeding starved patients, when carbohydrate administration reduces gluconeogenesis and lipolysis, in patients with sepsis, exogenous glucose and carbohydrates does not reduce the rates of gluconeogenesis and lipolysis(29).
2. The severity of TPN-induced hyperglycemia is directly proportional to the rate of glucose infusion and the degree of injury and age of the patients.
3. Starting enteral nutrition within 24 hours of ICU admission seems to improve intestinal glucose absorption, when compared with delayed enteral feeding (4 days after admission)(30,31).
4. Patients should receive 5% dextrose infusions (170 kcal/L), which enters the gluconeogenesis pathway.
5. Overfeeding glucose (>4mg/kg/min) and receiving a total caloric intake greater than the REE in patients with critical illness results in further elevation of blood glucose and production of additional CO2.
6. In critically ill patients, the rate and extent of enteral glucose absorption are reduced due to delayed gastric emptying along with reduced small intestinal absorption (32).
7. In critically ill patients, tight glucose control leads to moderate or severe hypoglycemia, both of which are associated with an increased mortality (33).
8. Poorly controlled hyperglycemia in critically ill patient is associated with increased morbidity and mortality(34). Hyperglycemia is associated with poor immune response, increased cardiovascular events and thrombosis.

## LIPIDS

1. Intravenous lipid is recommended to be administered as a long-chain TG (LCT) emulsion. These emulsions contain soybean oil and an emulsifier (egg phospholipid) and 10% and 20% solutions contain 1.0 and 2.0 kcal/mL, respectively.
2. The lipid emulsion is converted to TG-rich particles with sizes similar to that of chylomicrons and phospholipid-rich particles(35).
3. The current Iranian guideline for nutrition in ICU recommends considering lipid emulsions as 30% to 40% of total TPN calories as energy substrates.
4. Patients receiving TPN should receive lipid emulsion (500 mL of a 10% LCT emulsion) at a minimum of two times a week to prevent essential FFA deficiency.
5. Soybean-derived LCT emulsions traditionally used for TPN, have high proportions of omega-6, polyunsaturated fatty acids, specifically linoleic acid (53%). Linoleic acid is the precursor of thromboxane A2 and prostaglandin E1, which cause platelet aggregation and inflammation.
6. Alternatively, fish oils containing omega-3 fatty acids (e.g., eicosapentaenoic and linolenic acids) are precursors of different prostaglandins (e.g., thromboxane A3).
7. Formula containing both LCT and medium-chain TGs (MCTs) or mono-saturated fatty acids derived from olive oil is recommended to substitute LCT alone and have fewer side effects such as production of toxic lipid peroxides and immunosuppression(36).

## PROTEIN AND AMINO ACID

1. Amino acids and protein are considered as safe and favorable therapeutics (37, 38) as well as essential ingredients of nutritional support regimens. TPN contains commercially prepared amino acid mixtures, whereas enteral formulas contain free amino acids, peptides, or whole proteins.
2. Amino acids and protein are not well utilized since proteolysis is relatively unresponsive to the usual negative feedback mechanisms of administration of exogenous glucose, protein, or amino acids; therefore nitrogen balance remains negative in ICU.
3. In the catabolic state, protein–amino acid intake of 1.2 to 1.5 g/kg/day is recommended. Higher amounts do not promote further nitrogen retention and only blood urea nitrogen may rise.
4. Glucose and fat should be concomitantly administered as an energy source to ensure that the protein–amino acids are not metabolized.
5. Insulin at lower doses should be administered to decrease protein breakdown by inhibiting the major catabolic pathway(39).

## OTHER NUTRITIENTS

1. If Polymeric formula is used, as a result of formula variation, calories and protein needs are overestimated(40).
2. Glutamine and Selenium supplementations could be used for patient in ICU, considering their proven benefits in oxidative stress conditions like pulmonary diseases.
3. A summary of essential nutrients is provided in table 5.

**Table 5.** Daily Caloric Requirements

Nutrients	Daily Requirements	Supplement
Carbohydrate	2 g/Kg/day	5% Dextrose (170Kcal/L)
Lipid	0.7–1.5g/Kg/day	10% and 20% Soy Bean Oil (1 and 2 Kcal/ml)
Protein/Amino acid(AA)	1.2–1.5g/Kg/day	If TPN then free AA mixture should be used.
		If EN then free AA mixture, peptides or whole protein could be used.
Other	If required	Glutamine, Selenium

## METABOLIC AND PROCEDURAL COMPLICATIONS

1. Although morbidity is more related to enteral nutrition than parenteral nutrition; infectious complications are more common with parenteral nutrition.
2. Central venous catheterization could be complicated with pneumothorax.
3. **Hyperglycemia** is a common problem in critically ill patients, especially with severe stress, steroid use, and diabetes mellitus, requiring treatment with insulin.
4. Administering of insulin is preferable over reducing the glucose intake, unless hyperglycemia is excessive (>250 mg/dL) despite high insulin doses. However; patients may face clinical complication with hypokalemia and hypophosphatemia.
5. **Hypoglycemia** can occur on the abrupt discontinuation of continuous feedings containing significant amounts of carbohydrate due to increase in insulin concentrations after feeding.
6. Overfeeding patients with critical illnesses has many consequences depicted in Table 6.

## ENERGY REQUIREMENT IN THE CRITICALLY ILL CHILD

1. Energy consumption should be measured regularly in the intensive care unit (41).
2. Using formulas is not an accurate method to assess children in the intensive care unit (41).
3. In this group of patients the best method to evaluate the energy consumption is the indirect method of measuring calories (indirect calorimetry) (41).
4. Measurement of energy consumption in these critically ill children can determine the status of the child to see if the patient is in hyper or hypo metabolic state which is achieved by using indirect calorimetry in a daily basis (41).
5. This method is a reliable no-risk, non-invasive and reproducible method. Besides it is possible to use this method when the patient is connected to a ventilator
6. The indirect calorimetry method has its limitations. For example, pain and fever, and the use of some medications affect the energy consumption when using indirect calorimetry, which are ignored when using this method.
7. A simple alternative method is to replace indirect calorimetry with using a Douglas bag (42).
8. Since the basic energy measurement shows the minimum required energy, the question is how much energy is needed in acute and recovery phases. It is anticipated that the needed energy in the recovery phase for tissue regeneration (example: for wound healing) as well as the energy needed to induce growth is higher than basic energy. Almost four calories should be added to basic energy per gram of added weight (43).
9. Another parameter derived from indirect calorimetry is respiratory quotient. If this ratio is less than 0.85 it is a sign of poor nutrition, and if the ratio is more than one it is a sign of over nutrition (44,45).
10. Traditionally the stress level and activity level are used to calculate the exact needed energy. These variables change with the severity of disease and amount of patients’ activity. On the other hand critically ill patients might be connected to a mechanical ventilator and receive tranquilizer drugs. In these patients the activity is reduced, fluid excretion rate is diminished and the growth is limited in the active phase of disease, thus if the stress level or activity is included in calculation of needed energy the patient might receive excess energy which causes overweight. So it is not commonly suggested to routinely use the activity and stress factors when calculating the energy for patients in intensive care unit (41).

## ETHICAL ASPECTS OF ARTIFICIAL NUTRITION

The guidelines over nutrition in end-of life ICU admitted patients are controversial due to variability in cultures and religious believes in different countries. However, the scientific and medical facts in this regard are unequivocal. Nutrition and hydration are a medical intervention, requiring an indication, a therapeutic goal and the will (consent) of the competent patient (46).

**Table 6.** Consequences of Overfeeding in Parenteral and Enteral Nutrition

Consequences of Overfeeding in Parenteral and Enteral Nutrition	
**Caloric intake (**>1.5–1.7 REE in patients with critical illnesses)	Increased fat storage, CO2 production, urea production
**Carbohydrates** (>4.5 g/kg/min)	poor immune response, increased cardiovascular events, thrombosis
	**Lungs**: Increased CO2 production
	**Fatty liver:** Hepatomegaly, increases AST, ALT, and alkaline phosphatase.
	**Hyperglycemia:** osmotic diuresis
	*Reduces insulin sensitivity*
	*Increased intracellular potassium:*
	Decreases serum potassium.
	*Increased intracellular phosphate:*
	Decreases serum phosphate.
**Lipids (>**2g/kg/day)	**Liver**: Cholestasis, fatty liver, increase Serum triglyceride
**Protein(**>2g/kg/day)	**Kidneys**: Ureagenesis is increased.
	BUN increases with decreased renal function.
**Fluid intake**	fluid overload

ICU is where patients are being cared for by technologically advanced life-sustaining treatments, and where difficult decisions are made about the usefulness of such treatments (47). Despite the special consideration given to the end of life issues in critically ill patients, the decision to withhold and withdraw life-sustaining treatment in this patient population remains a challenge even when there is minimum chance of recovery.

In Iran, the medical ethics is based on both medical aspects and Islamic laws. From the perspective of Islam, nutritional support is considered as a basic an ethical care, not as a medical treatment. In Islamic law and tradition, the withholding or withdrawing of nutrition or hydration resulting in starvation, is seen as a greater harm than any potential complications of that treatment. To this end it is advised that starvation has to be avoided in such situations (42, 48).
